# Exogenous Pancreatic Kallikrein Improves Diabetic Cardiomyopathy in Streptozotocin-Induced Diabetes

**DOI:** 10.3389/fphar.2018.00855

**Published:** 2018-08-07

**Authors:** Meng Wu, Yeping Yang, Meng Wang, Fangfang Zeng, Qin Li, Wenjuan Liu, Shizhe Guo, Min He, Yi Wang, Jie Huang, Linuo Zhou, Yiming Li, Ji Hu, Wei Gong, Zhaoyun Zhang

**Affiliations:** ^1^Division of Endocrinology and Metabolism, Huashan Hospital, Fudan University, Shanghai, China; ^2^Department of Endocrinology, The Second Affiliated Hospital, Soochow University, Suzhou, China; ^3^Division of Endocrinology and Metabolism, Shanghai Ninth People’s Hospital, Shanghai Jiao Tong University School of Medicine, Shanghai, China; ^4^Institute of Endocrinology and Diabetology, Fudan University, Shanghai, China; ^5^Changzhou Qianhong Biopharma Co., Ltd., Changzhou, China

**Keywords:** pancreatic kallikrein, diabetic cardiomyopathy, kallikrein-kinin system, fibrosis, inflammation, oxidative stress, Ca^2+^-handling protein

## Abstract

**Aims:** To evaluate the protective effects of exogenous pancreatic kallikrein (PKK) treatment on diabetic cardiomyopathy (DCM) and explore the underlying mechanisms.

**Methods and Results:** Streptozotocin (STZ)-induced diabetic rats, a type 1 diabetic model, were treated with either PKK or saline for 12 weeks. Non-diabetic rats were used as controls. PKK administration attenuated the mitochondria swelling, Z line misalignments, myofibrosis and interstitial collagen accumulation in diabetic myocardial tissue. The oxidative stress imbalance including increased nitrotyrosine, decreased anti-oxidative components such as nuclear receptor nuclear factor like 2 (Nrf2), glutathione peroxidase 1(GPx-1), catalase (CAT) and superoxide dismutase (SOD), were recovered in the heart of PKK-treated diabetic rats. In diabetic rats, protein expression of TGF-β1 and accumulation of collagen I in the heart tissues was decreased after PKK administration. Markers for inflammation were decreased in diabetic rats by PKK treatment. Compared to diabetic rats, PKK reversed the degradation of IκB-α, an inhibitive element of heterotrimer nuclear factor kappa B (NF-κB). The endothelial nitric oxide synthase (eNOS) protein and myocardial nitrate/nitrite were impaired in the heart of diabetic rats, which, however, were restored after PKK treatment. The sarcoplasmic reticulum Ca^2+^-ATPase 2 (SERCA2) and phospholamban (PLN) were mishandled in diabetic rats, while were rectified in PKK-treated diabetic rats. The plasma NT-proBNP level was increased in diabetic rats while was reduced with PKK treatment.

**Conclusion:** PKK protects against DCM via reducing fibrosis, inflammation, and oxidative stress, promoting nitric oxide production, as well as restoring the function of the calcium channel.

## Introduction

Diabetic cardiomyopathy (DCM) was first introduced by Rubler et al. (1972), who presented autopsy data from four diabetic patients. Currently, DCM is defined as an abnormal myocardial structure without pathological changes such as coronary heart disease, congenital heart diseases, valvular heart disease, or hypertension (Jia et al., 2016). DCM is a main pathogenic factor for heart failure in diabetic patients (Kannel et al., 1974; Devereux et al., 2000). Among patients with DCM, the cumulative probability of death was 18%, and development of heart failure was 22% (Kain and Halade, 2017). In Framingham study, the heart failure rates in men and women with diabetes were both significantly higher than that in people without diabetes (Sung et al., 2015). So far, therapy for patients with diabetes focuses largely on glucose control. Before the onset of heart failure symptoms, patients lack enough standard treatments (Sung et al., 2015; Kain and Halade, 2017). Nevertheless, there remains a significant incidence of cardiovascular disease even in optimally treated diabetic patients (Tate et al., 2017). Therefore, more effective strategies are required to specifically target DCM.

Several alterations have been proposed to explain the mechanism of DCM, including oxidative stress, myocardial inflammation, increased deposition of collagen in myocardial interstitium, and impaired calcium homeostasis (Boudina and Abel, 2010; Kovacic et al., 2014). Thus, the ideal therapy would be targeting these multiple pathological pathways. The kallikrein-kinin system (KKS) is involved in multiple physiological and pathophysiological aspects including oxidative stress, remodeling, and inflammation (Chao et al., 2006). Our previous research reported that exogenous pancreatic kallikrein (PKK) could improve diabetic nephropathy through decreasing oxidative stress, fibrosis and inflammation (Liu W. et al., 2016). Therefore, we aimed to explore whether exogenous PKK supplementation could also protect against DCM. We found that PKK treatment could ameliorate the pathological alterations and improve the function of DCM, which might be mediated by decreasing myocardial oxidative stress, inflammation, and interstitial fibrosis as well as by improving Ca^2+^-handling proteins in the heart.

## Materials and Methods

### Animal Study

Our animal experiments were conducted with the permission of the Institutional Animal Care and Use Committee of Fudan University. The Guide for the Care and Use of Laboratory Animals published by the Chinese National Institutes of Health were followed during the study. Five-week-old male Sprague-Dawley (SD) rats were provided by the SLRC Laboratory Animal Center (Shanghai, China). Type 1 diabetic rats were induced by a single-dose of intraperitoneally injected streptozotocin (STZ, 65 mg/kg in 0.1 mol/L citrate buffer solution, pH 4.5; Sigma, MO, United States). An equivalent volume of citrate buffer vehicle was administered to the non-diabetic group. Diabetes was defined as blood glucose levels of 16.7 mmol/L 1 week after STZ injection. Random blood glucose and body weight were checked weekly and blood pressure was measured by tail-cuff plethysmography monthly (BP-98A, Softron Beijing Incorporated, Beijing, China). After the onset of diabetes, diabetic rats were allocated into two groups (*n* = 6 in each group). One group (DM + PKK group) was treated with daily peritoneal injection of PKK (60 U/kg/d; Qianhong Bio-pharma Company, Changzhou, China), and the control group (DM group) was treated with saline (0.1 ml/10 g/d). The dose of PKK used in this study was based on previous study (Liu W. et al., 2016). Non-diabetic control rats (NDM group) received the same saline injection as the DM group (0.1 ml/10 g/d). Rats were housed in a temperature-controlled room, maintained under a 12-h light/12-h dark cycle and were provided free access to regular water and standard laboratory chow. Before sacrifice, rats were anesthetized with intraperitoneal injection of 10% chloral hydrate (0.3 ml/100 g). The adequacy of anesthesia was determined by the loss of a pedal withdrawal reflex (Chen et al., 2013). After being treated for 12 weeks, all animals were sacrificed. Hearts were excised immediately and washed thoroughly with saline to flush the blood. Heart tissues were isolated for further analysis.

### Transmission Electron Microscope

Left ventricular samples for electron microscopy were cut into approximately 1 mm^3^ pieces and fixed in 10% glutaraldehyde overnight. After dehydration and embedding as described previously (Hou et al., 2013), ultrathin sections were cut from blocks and mounted on copper grids. The grids were then counterstained with lead citrate and uranylacetate. The images were recorded under a transmission electron microscope (FEI Tecnai G2 Spirit, Hillsboro, OR, United States).

### Histologic Evaluation

Left ventricular myocardium were fixed in 4% paraformaldehyde overnight. Tissues were embedded in paraffin, stained with hematoxylin and eosin (H&E), PAS, or Masson reagent as described previously, and examined using an optical microscope (x400, Olympus, Richmond Hill, ON, Canada). Myocyte area was determined perpendicularly to the outer contour of the cell membrane at the nucleus level. The cross-sectional area of single myocytes was measured using ImageJ software. The outline of 100–200 cardiomyocytes was traced in each group. Average myocyte area of each sections was calculated. For the quantification of PAS and Masson staining, at least 10 images were captured for each section. ImageJ was used for the analysis and the results were expressed as the percentage of PAS (or Masson) staining positive area out of the total area of the cross-section.

### Immunohistochemistry Analysis

Heart tissues were fixed with 4% paraformaldehyde overnight and then embedded in paraffin. Expressions of nitrotyrosine, TNF-α, TGF-β1, and collagen I were examined on the tissue section using immunohistochemical (IHC) analyses as described previously (Tian et al., 2009). The dilution concentration of nitrotyrosine antibody (Millipore, Burlington, MA, United States), tumor necrosis factor α (TNF-α) antibody (Abcam, Cambs, United Kingdom), transforming growth factor β1 (TGF-β1) antibody (Abcam, Cambs, United Kingdom), collagen I antibody (Abcam, Cambs, United Kingdom), and CD31 (Abcam, Cambs, United Kingdom) was 1:100, 1:50, 1:200, and 1:300, 1:500, respectively. For IHC analysis, each section was captured in at least 10 pictures and all pictures were quantified. The protein expression intensity was assessed by estimating the area of the objects and the medium pixel intensity per object, as the integrated optical density (IOD). IOD to area ratio was used to present the results. All images were acquired and processed in TIFF format, analysis was done using Image Pro Plus 6.0 software (Media Cybernetics, Rockville, MD, United States). Capillary density was assessed by capillaries/myocyte nucleus (C/M) values. C/M values were counted in 5 fields randomly selected from each slice.

### RNA Extraction and Real-Time Polymerase Chain Reaction (Real-Time PCR)

RNA was extracted from the heart tissue using Trizol reagent (Life Technologies, Waltham, MA, United States). RNA concentration was determined in a spectrophotometer (Nanodrop 2000c, Thermo Scientific, Waltham, MA, United States) at the absorbance of 260 nm. Then, 2 μg of total RNA was converted to cDNA according to the manufacturer’s protocol (G490, Abm, Canada). Target cDNA was analyzed for the expression of genes with the ABI 7500 Sequence Detection System (Applied Bio-systems, Waltham, MA, United States) using the following conditions: 94°C, 5 min; 94°C, 30 s, 55°C, 30 s, 72°C, 1 min 30 s, 40 cycles; 72°C, 10 min. The levels of target gene expression were quantified using a complementary DNA standard curve and data were normalized to β-actin or GAPDH. The results were expressed as fold change. The primers used are shown in **Table 1** and **Supplementary Table 1**. Each sample was repeated for three times.

**Table 1:** Sequences of primers used for real-time PCR.

	Forward	Reverse
GPx-1	5′-GACCGACCCCAAGTACATCA-3′	5′-GCAGGGCTTCTATATCGGGT-3′
GR	5′-AAGCACTTCTCACCCCAGTT-3′	5′-CGGCTTCATCTTCAGTGAGC-3′
CAT	5′-CCTCAGAAACCCGATGTCCT-3′	5′-TCAGGAATCCGCTCTCTGTC-3′
SOD	5′-GGCCAAGGGAGATGTTACAA-3′	5′-GCTTGATAGCCTCCAGCAAC-3′
Nrf2	5′-TGTCAGCTACTCCCAGGTTG-3′	5′-ATCAGGGGTGGTGAAGACTG-3′
TGF-β1	5′-CCTGCAAGACCATCGACATG-3′	5′-TGTTGTACAAAGCGAGCACC-3′
Collagen I	5′-TCAAGATGGTGGCCGTTACT-3′	5′-CATCTTGAGGTCACGGCATG-3′
TNF-α	5′-TCATCCGTTCTCTACCCAGC-3′	5′-TACTTCAGCGTCTCGTGTGT-3′
MCP-1	5′-ACCAGCCAACTCTCACTGAA-3′	5′-GCCAGTGAATGAGTAGCAGC -3′
CD68	5′-GGAATGCCACAGTTTCTCCC-3′	5′-CTGAACACATGGCTGGGAAC-3′
IL-6	5′-CTCATTCTGTCTCGAGCCCA-3′	5′-CTGTGAAGTCTCCTCTCCGG-3′
β-actin	5′-CTGGAGAAACCTGCCAAGTATGAT-3′	5′-TTCTTACTCCTTGGAGGCCATGTA-3′
GAPDH	5′-ACCACAGTCCATGCCATCAC-3′	5′-TGCCAGTGAGCTTCCCGTT-3′

*GPx-1, glutathione peroxidase-1; GR, glutathione reductase; CAT, catalase; SOD, superoxide dismutase; TGF-β1, transforming growth factor β1; TNF-α, tumor necrosis factor α; MCP-1, monocyte chemo-attractant protein 1; Nrf2, nuclear factor erythroid 2-related factor 2; CD68, cluster of differentiation 68; IL-6, interleukin-6; eNOS, endothelial nitric oxide synthase.*

### Protein Extraction and Western Blot Analysis

The heart tissues were homogenized in an ice-cold lysis buffer. These protein samples (30 μg) were resolved using SDS-polyacrylamide gel (10%) electrophoresis and transferred to PVDF membranes (Millipore, Burlington, MA, United States). The membranes were blocked with 5% non-fat milk for 1 h at room temperature and incubated with rabbit anti-collagen I polyclonal antibody (1:5000, Abcam, Cambridge, MA, United States), rabbit anti-TGF-β1 polyclonal antibody (1:2000, Abcam), rabbit anti-IκB-α monoclonal antibody (1:2000, Abcam), rabbit anti-GAPDH antibody (1:5000, Abcam), mouse anti-sarcoplasmic reticulum Ca^2+^-ATPase 2 (SRECA2) monoclonal antibody (1:500, Santa Cruz Biotechnology, Dallas, TX, United States), mouse anti-phospholamban (PLN) monoclonal antibody (1:500, Santa Cruz Biotechnology), mouse anti-endothelial nitric oxide synthase (eNOS) monoclonal antibody (1:500, Santa Cruz Biotechnology), rabbit anti-bradykinin receptor type I (B1R) monoclonal antibody (1:500, Bioss, Beijing, China), rabbit anti-bradykinin receptor type I (B1R) monoclonal antibody (1:500, Bioss, Beijing, China), or rabbit anti-bradykinin receptor type II (B2R) monoclonal antibody (1:500, Santa Cruz Biotechnology), washed and incubated with goat anti-rabbit peroxidase-coupled secondary antibodies (1:5000, Cell Signaling Technology, Danvers, MA, United States) or goat anti-mouse peroxidase-coupled secondary antibodies (1:5000, Cell Signaling Technology). The blots were visualized with an enhanced chemiluminescence reaction (Perkin Elmer Life Science, Waltham, MA, United States) (Zhang et al., 2010). Each sample was repeated for three times.

### Measurement of Serum Lipids

Serum triglyceride (TG), total cholesterol (TC), low density lipoprotein cholesterin (HDL-C) and high density lipoprotein cholesterin (LDL-C) was measured with a spectrophotometric method according to the manufacturer’s instructions (A110-1, A111-1, A112-1, A113-1; Nanjing Jiancheng Bioengineering Institute, Nanjing, China).

### Measurement of Myocardial Glutathione (GSH) and Glutathione Disulfide (GSSG)

Myocardial GSH and GSSG were measured with a spectrophotometric method according to the manufacturer’s instructions (703002, Cayman Chemicals, Ann Arbor, MI, United States).

### Measurement of Total Nitrate/Nitrite Concentration in Heart Tissues

Myocardial nitrate/nitrite concentration was determined using a spectrophotometric method according to the manufacturer’s instructions (780001, Cayman Chemicals, Ann Arbor, MI, United States).

### Measurement of Plasma N-terminal Protein B-type Natriuretic Peptide

Whole blood was collected into tubes containing EDTA as an anticoagulant and was centrifuged for 15 min at 3000 rpm. Plasma was decanted and preserved as single aliquots and stored in a freezer set to maintain -80 °C. N-terminal protein B-type natriuretic peptide (NT-proBNP) was analyzed according to the manufacturer’s instructions (E08752r, CUSABIO, SH, China).

### Statistical Analysis

All data were presented as mean ± SEM from three independent experiments. Statistical analysis was performed with the statistical package SPSS for Mac Ver. 20.0 (SPSS, Inc., Chicago, IL, United States). The significance of the differences in mean values among different groups was evaluated using one-way ANOVA followed by Tukey’s test. *P*-values less than 0.05 were considered statistically significant. All graphs were constructed using Prism program (GraphPad, La Jolla, CA, United States).

## Results

### Basic Characteristics of Rats

Type 1 diabetic rats from the DM group developed robust, sustained, and equivalent hyperglycemia and lower body weight compared to the NDM group. There was no difference in both systolic and diastolic blood pressures between the NDM group and the DM groups. Additionally, exogenous PKK had no effect on the blood glucose, serum lipids, body weight, and blood pressures in STZ-induced diabetic rats (**Table 2**).

**Table 2:** Basic characteristics of SD rat.

	NDM (*n* = 6)	DM (*n* = 6)	DM + PKK (*n* = 6)	*P*-values
BW (g)	527.10 8.61	218.90 7.65^∗^	216.7 12.85^#^	<0.05
BG (mmol/l)	6.26 0.32	32.69 0.41^∗^	30.50 1.14^#^	<0.05
TG (mmol/l)	1.43 0.13	2.18 0.49	2.52 0.73	>0.05
TC (mmol/l)	2.15 0.18	2.29 0.17^∗^	2.80 0.46	<0.05
LDL-C (mmol/l)	0.34 0.04	0.51 0.05^∗^	0.68 0.12	<0.05
HDL-C (mmol/l)	0.83 0.05	1.21 0.09^∗^	1.41 0.08	<0.05
SBP (mmHg)	136.10 1.82	147.50 5.38	159.1 6.44	>0.05
DBP (mmHg)	81.13 5.25	81.43 5.39	85.33 11.37	>0.05

*BW, body weight; BG, blood glucose; SBP, systolic blood pressure; DBP, diastolic blood pressure. Data are means ± SEM. ^∗^P < 0.05 vs. NDM group, ^#^P < 0.05 vs. DM group.*

### PKK Administration Improved the Cardiac Ultrastructure of Diabetic Rats

**FIGURE 1:**
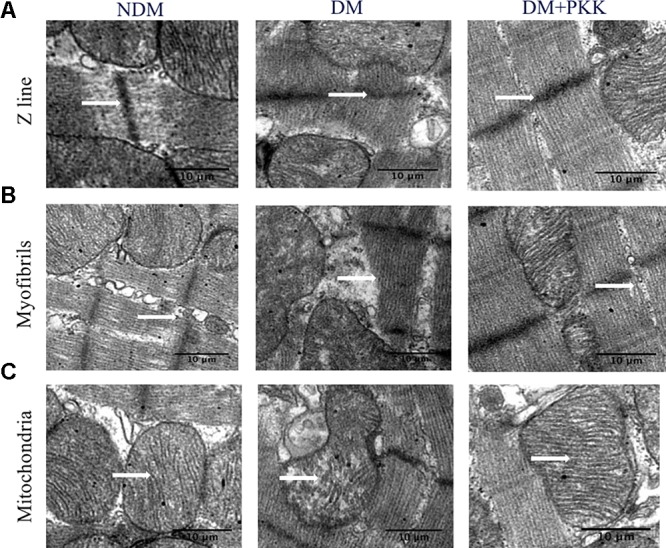
Pancreatic kallikrein administration improved the ultrastructure of diabetic cardiomyopathy in STZ-induced diabetic rats. The ultra-microstructures for arrangement of **(A,B)** myocardial fibers and **(C)** mitochondria. In the DM group, myocardial fibers, as well as mitochondria, were misarranged. Irregular arrangement of myofibrils was observed and Z lines were obscured. The mitochondrial structures showed signs of swelling and breakage of cristae. White arrows represent Z lines, myofibrils or mitochondria. However, pancreatic kallikrein (PKK) treatment reversed these pathological changes compared with the DM group.

Compared with the NDM group, the adjacent sarcomere displayed Z line misalignments (**Figure 1A**, white arrows) and disruptions in the DM group. The myofibrils were irregularly arranged (**Figure 1B**, white arrows). The mitochondria swelled, and the mitochondrial cristae were irregular. Some cristae were fractured in the myocytes from the left ventricular myocardium in the diabetic rats (**Figure 1C**, white arrows). However, these pathological changes of cardiac ultrastructure in diabetic rats were prevented by PKK treatment.

### PKK Administration Protected Against Myocardial Oxidative Stress in STZ-Induced Diabetic Rats

**FIGURE 2:**
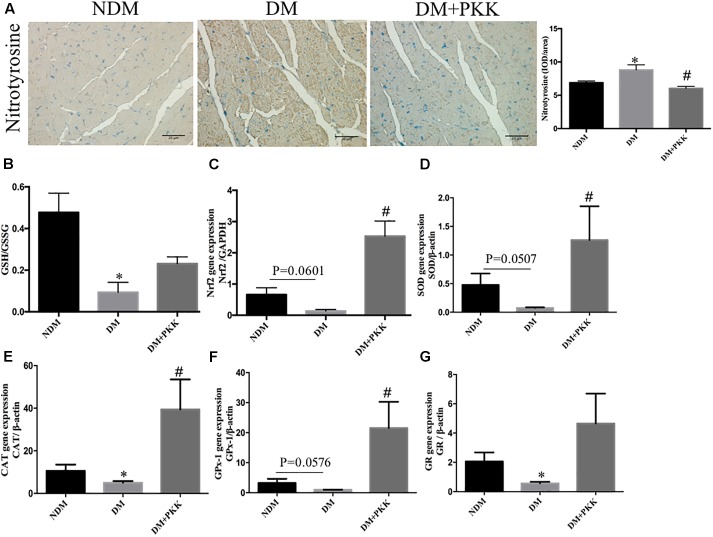
Pancreatic kallikrein administration protected against myocardial oxidative stress in STZ-induced diabetic rats. **(A)** Representative IHC micrographs of myocardium tissues stained with nitrotyrosine for the NDM, DM, and DM + PKK groups (original magnification × 40). The IHC scores of myocardium sections were quantified. **(B)** Myocardial GSH/GSSG ratio. **(C–G)** The mRNA expression of markers related with oxidative stress in heart tissues. **(C)** Nrf2, **(D)** SOD, **(E)** CAT, **(F)** GPx-1, and **(G)** GR. Each sample for qPCR was repeated for three times. Values are mean ± SEM, *n* = 6 per group; ^∗^*P* < 0.05 vs. NDM group; ^#^*P* < 0.05 vs. DM + PKK group.

As shown in **Figure 2A**, we measured myocardial oxidative stress using immunohistochemistry staining of nitrotyrosine, which is an indicator of oxidative stress. Compared with the NDM group, the accumulation of nitrotyrosine was increased in the myocardial tissues of diabetic rats, which was reduced by PKK treatment (**Figure 2A**). The GSH/GSSG ratio represents the cellular capacity of anti-oxidative stress. Compared to the NDM group, the cardiac GSH/GSSG was significantly decreased in the DM group, which was increased by PKK treatment (DM group vs. DM+PKK group, *p* = 0.0561) (**Figure 2B**). In addition, we also examined the gene expression of Nrf2, SOD, CAT, GR and GPx-1 (**Figures 2C–G**), which are major anti-oxidan components. All the mRNA expression of these components was decreased in the DM group, which was ameliorated following PKK treatment.

### PKK Administration Protected Against Myocardial Hypertrophy and Fibrosis in the Diabetic Rats

**FIGURE 3:**
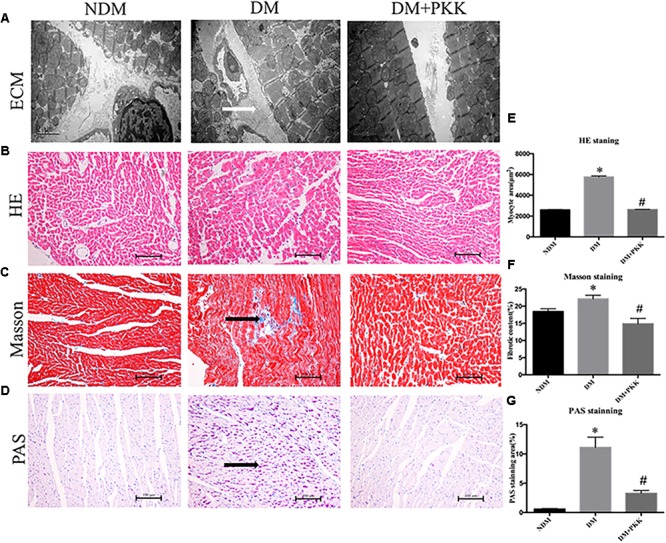
Pancreatic kallikrein administration protected against myocardial hypertrophy and fibrosis in the diabetic rats. **(A)** Representative transmission electron microscopy micrographs of myocardium tissues. White arrow indicates the accumulated collagen in the myocardial matrix. Representative images of cardiac tissue stained with **(B)** hematoxylin and eosin, **(C)** Masson (black arrow indicates the accumulated collagen in the myocardial matrix), and **(D)** PAS (black arrow indicates the accumulated glycogen in cardiomyocytes) for the NDM, DM, and DM + PKK groups (original magnification × 40). **(E)** Quantitative results of the cross-sectional diameter of myocytes within transverse cardiac sections. **(F,G)** Quantitative results of the cardiac extracellular matrix and collagen accumulation with Masson and PAS for the NDM, DM, and DM + PKK groups are presented (original magnification x40). Values are mean ± SEM, *n* = 6 per group; ^∗^*P* < 0.05 vs. NDM group; ^#^*P* < 0.05 vs. DM + PKK group.

In **Figure 3A**, the DM group displayed the myocardial accumulation of interstitial collagen (white arrows), whereas it was seldomly observed in the NDM group and DM+PKK group.

We used H&E, PAS, and masson staining to observe the myocardial pathological changes. We found that in the DM group, the cardiomyocyte showed hypertrophy compared to the NDM group, which was improved following PKK treatment (**Figures 3B,E**). Masson (**Figures 3C,F**) and PAS (**Figures 3D,G**) staining detected more collagen and glycogen, respectively, in the myocardial interstitium of the DM group, compared to both NDM and DM+PKK groups (**Figures 3C,D,F,G**). Black arrows represent the accumulated collagen and glucogen, respectively.

**FIGURE 4:**
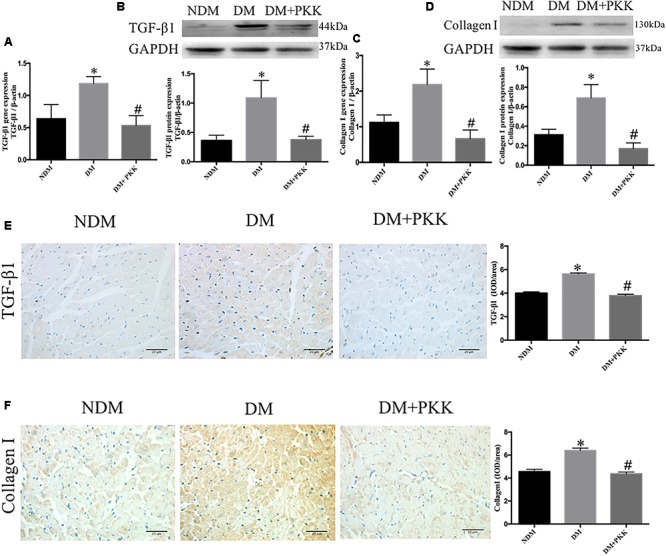
Pancreatic kallikrein administration reduced the expression of transforming growth factor β1 and collagen I in DCM. **(A)** The mRNA expression of TGF-β1. **(B)** The protein expression and quantitation of TGF-β1 using western blot. **(C)** The gene expression of collagen I. **(D)** The protein expression and quantitation of collagen I using western blot. **(E,F)** Representative IHC micrographs of cardiac tissue stained with TGF-β1 and collagen I for the NDM, DM, and DM + PKK groups (original magnification × 40). The IHC quantitative data of heart sections for TGF-β1 and collagen I staining are summarized. Each sample for qPCR and western blot was repeated for three times. Values are mean ± SEM, *n* = 6 per group; ^∗^*P* < 0.05 vs. NDM group; ^#^*P* < 0.05 vs. DM + PKK group.

Furthermore, both the mRNA (**Figures 4A,C**) and protein levels (**Figures 4B,D–F**) of TGF-β1 as well as collagen I were increased in the diabetic hearts, which were significantly reversed in the DM + PKK group.

### PKK Administration Abolished the Inflammation of Myocardial Tissues in Diabetic Rats

**FIGURE 5:**
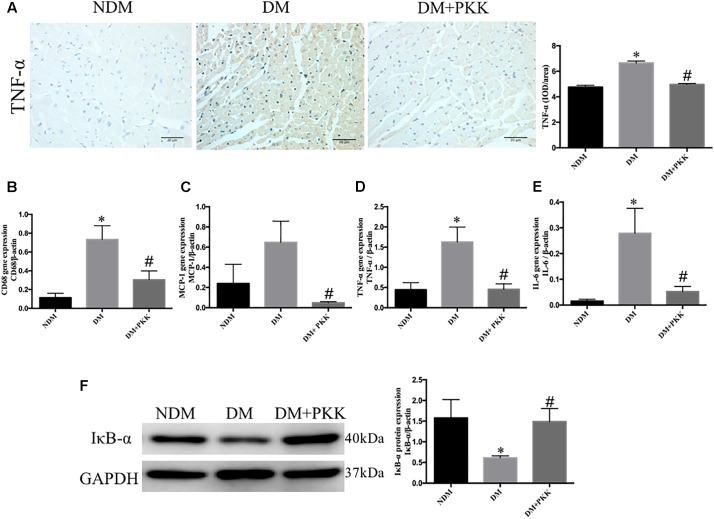
Pancreatic kallikrein administration abolished myocardial inflammation in STZ-induced diabetic rats. **(A)** The IHC micrographs and quantitative data of myocardium tissue stained with TNF-α in the NDM, DM, DM + PKK groups (original magnification 40×). The mRNA expression of **(B)** CD68, **(C)** MCP-1, **(D)** TNF-α, and **(E)** IL-6. **(F)** The protein expression and quantitative data of IκB-α. Each sample for qPCR and western blot was repeated for three times. Values are mean ± SEM, *n* = 6 per group; ^∗^*P* < 0.05 vs. NDM group; ^#^*P* < 0.05 vs. DM + PKK group.

As inflammation was involved in the development of DCM (Liao et al., 2017; Wang et al., 2018), we evaluated the expression of tumor necrosis factor (TNF-α) using IHC staining (**Figure 5A**). The protein levels of TNF-α in the diabetic hearts was increased compared with the NDM group, whereas it was ameliorated in the DM+PKK group. We also detected the mRNA expression of inflammatory markers, including clusters of differentiation 68 (CD68), monocyte chemo-attractant protein 1 (MCP-1), TNF-α, and interleukin-6 (IL-6) (**Figures 5B–E**). Expression of these proinflammatory markers was upregulated in the diabetic heart, with a similar trend evident for TNF-α protein, while PKK administration reversed that significantly. Activation of the NF-κ B pathway plays an important role in the induction of myocardial inflammation (Liu X. et al., 2016). We assessed IκB-α, a major inhibitor of NF-κB (Karin and Ben-Neriah, 2000). IκB-α was decreased in diabetic hearts, while was increased by PKK treatment (**Figure 5F**).

### PKK Administration Restored the Expression of Ca^2+^-Handling Proteins and Cardiac Function of Diabetic Rats

**FIGURE 6:**
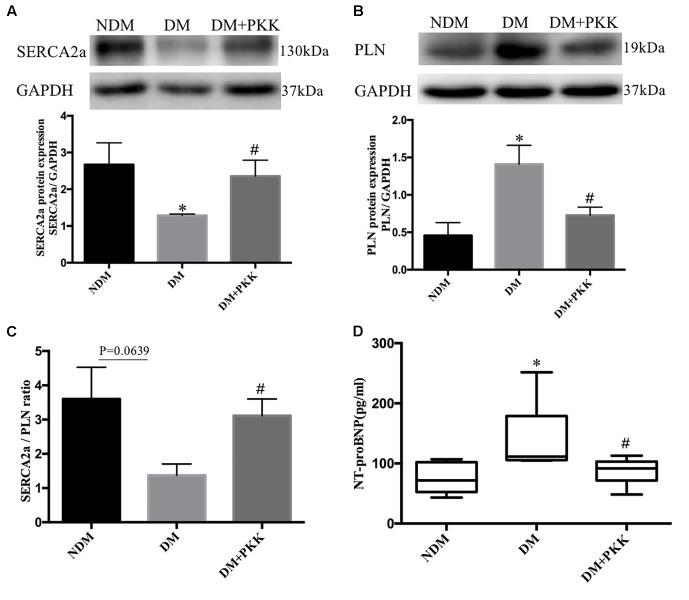
Pancreatic kallikrein administration restored the expression of Ca^2+^-handling proteins in the heart of STZ-induced diabetic rats. **(A,B)** The protein expression and the quantitative data of SERCA2 and PLN examined using western blot. **(C)** The SERCA2/PLN ratio in myocardial tissues. **(D)** NT-proBNP plasma levels in the DM group were higher than in the NDM group and DM + PKK group. Each sample for western blot was repeated for three times. Values are mean ± SEM, *n* = 6 for per group; ^∗^*P* < 0.05 vs. NDM group; ^#^*P* < 0.05 vs. DM + PKK group.

Sarcoplasmic reticulum Ca^2+^-ATPase 2a (SERCA2a) is directly linked to pump cytosolic Ca^2+^ from the cardiomyocyte back into the sarcoplasmic reticulum (SR) during relaxation of the heart and improving cardiac function during heart failure (Kho et al., 2011; Wahlquist et al., 2014). In the DM group, the protein expression of SERCA2a was decreased compared with the NDM group, whereas it was increased after PKK treatment (**Figure 6A**). Phospholamban (PLN) is an inhibitor of myocardial SERCA2a, and dephosphorylated PLN interacts with SERCA2a and inhibits cardiac pumping activity (Haghighi et al., 2014). Compared to non-diabetic rats, the dephosphorylated PLN protein level was upregulated in DM rats, whereas PKK treatment down-regulated the dephosphorylated PLN in the heart of diabetic rats (**Figure 6B**). Therefore, PKK treatment increased the SERCA2a/PLN ratio (**Figure 6C**), suggesting improved cardiac relaxation and diastolic function by PKK treatment. Brain natriuretic peptide (BNP) is released from the ventricular cardiomyocytes in response to pressure and volume overload. N-terminal protein B-type natriuretic peptide (NT-proBNP) is a metabolite from BNP, which is a reliable biomarker well correlated with echocardiographic indices (Omland et al., 1996; Dong et al., 2006; Ceyhan et al., 2008; Palazzuoli et al., 2010), such as left ventricular ejection fraction (LVEF) and left ventricular end diastolic pressure (LVEDP). Therefore, we measured the plasma NT-proBNP level to evaluate the heart function. The concentrations of plasma NT-proBNP in the NDM, DM, and DM + PKK groups were 74.70 ± 8.596 pg/mL, 139.9 ± 23.64 pg/mL, and 86.40 ± 8.103 pg/mL, respectively (NDM group vs. DM group, *p* = 0.0116; DM vs. DM + PKK group, *p* = 0.0429). PKK decreased the plasma NT-proBNP in diabetic rats (**Figure 6D**), indicating that exogenous PKK could improve the heart function in STZ-induced diabetic rats.

### PKK Administration Promoted NO Production in the Heart of STZ-Induced Diabetic Rats

**FIGURE 7:**
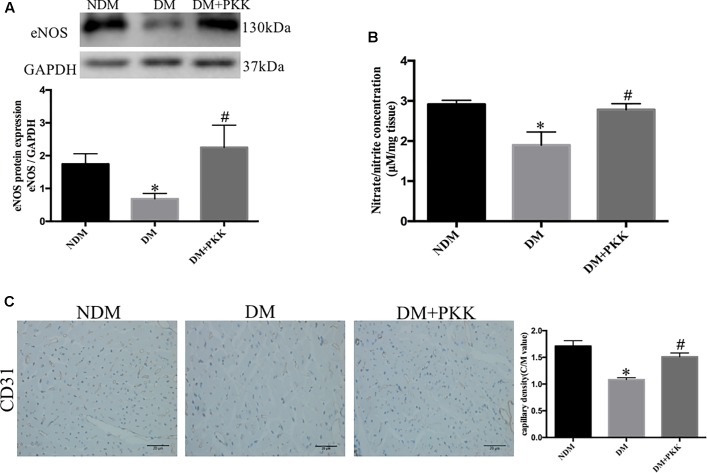
Pancreatic kallikrein administration promoted nitric oxide production in the heart of STZ-induced diabetic rats. **(A)** The protein expression and quantitative data of anti-endothelial nitric oxide synthase (eNOS) in the NDM, DM, and DM + PKK groups. **(B)** Myocardial nitrate/nitrite concentrations in diabetic rats were lower than that in the NDM group and DM + PKK group. **(C)** CD31 staining in NDM, DM, and DM + PKK group, respectively; and the comparison of C/M values among three groups. The magnification of light microscope is 40. The brown staining indicates the CD31+ capillary endothelial cells. Myocardial sections were counterstained with hematoxylin (the blue nucleus staining). Each sample for qPCR and western blot was repeated for three times. Values are mean ± SEM, *n* = 6 per group; ^∗^*P* < 0.05 vs. NDM group; ^#^*P* < 0.05 vs. DM + PKK group.

The eNOS/NO pathway is of importance in the progression of DCM and myocardial ischemia/reperfusion injury in diabetes (Paz et al., 2003). The endothelial dysfunction has been shown to be involved in diabetic microangiopathy and cardiomyopathy (Joshi et al., 2014). To determine whether the protective effects of PKK was mediated by this pathway, we measured the expression of eNOS and nitrite (NO^2-^)/nitrate (NO^3-^) in the heart homogenates. We found reduced eNOS (**Figure 7A**) and myocardial nitrite/nitrate (**Figure 7B**) in diabetic heart, suggesting reduction of NO production and the existence of microcirculation disturbance. PKK supplementation increased the protein expression of eNOS and myocardial nitrite/nitrate concentration in diabetic rats, which suggested that the beneficial effects of PKK on heart might be mediated by improving NO production and microcirculation.

To detect the diabetic microangiopathy and determine whether PKK reduces microangiopathy in the heart, we also measured CD31, a marker of capillary density, with immunohistochemistry. CD31 expression was decreased in diabetic hearts (NDM vs. DM: 1.711 ± 0.1021 vs. 1.083 ± 0.0359, *P* < 0.05), while was increased in DM + PKK group (DM vs. DM + PKK: 1.083 ± 0.03592 vs. 1.510 ± 0.0722, *P* < 0.05, **Figure 7C**). Therefore, PKK treatment might reduce the microangiopathy in DCM.

### KKS in the Myocardial Tissues of STZ-Induced Diabetic Rats

To examine the expression of the components of KKS, including kallikrein1, B1R and B2R, we performed RT-PCR. Compared with NDM group, kallikrein 1 was decreased in DCM while was significantly increased in DM + PKK group (**Supplementary Figure 1A**). Compared to NDM group, the expression of B1R mRNA was increased in DM group, while the expression of B2R mRNA was unchanged. There were no changes for both B1R and B2R mRNA and protein in DM + PKK group, compared to DM group (**Supplementary Figures 1B–F**).

## Discussion

Streptozotocin -induced diabetic rats, a well-established type 1 diabetic model for the study of DCM (Van Linthout et al., 2008; Lei et al., 2013; Shida et al., 2014; Ramirez-Correa et al., 2015) were used in our study. Several other methods have been used to induce diabetes in the rodents. Genetic models avoid the potential confounding effects of toxins, such as the Ins2+/- Akita diabetic mouse, ob/ob mouse and db/db mouse. Akita diabetic mouse is a type 1 diabetes model while the other two are type 2 models. Akita diabetic model has rarely been used for studies about DCM, while db/db mice have been used to study DCM. However, in our previous study which treated db/db mice with PKK, we found that PKK supplementation decreased triglyceride (Liu W. et al., 2016), which may also contribute to the protection of DCM. To exclude the effects of PKK on lowering triglyceride, here we used STZ-induced type 1 diabetes. And indeed, in STZ-induced type 1 diabetic rats, PKK showed no effects on serum lipids level including triglyceride. The loss of the body weight of diabetic rats may be attributed to the insulin deficiency and the long duration of hyperglycemia. PKK did not improve beta cell function and showed no effect on high glucose in diabetic rats. Therefore, PKK supplementation had no effect on reducing the loss of body weight and high blood glucose.

Pancreatic kallikrein has been used to treat diabetic microvascular complications for more than 30 years in China. However, little research has been done to investigate the mechanisms. Recently, we demonstrated that PKK possesses a variety of pharmacologic effects including anti-inflammation, anti-oxidative stress, and anti-fibrosis in diabetic nephropathy (Liu W. et al., 2016). As these pathological processes have been shown to be involved in the development of DCM, we speculated that PKK might also protect against DCM. Indeed, our study showed that exogenous treatment with PKK could prevent against DCM through ameliorating inflammation, oxidative stress and fibrosis, stimulating the production of nitric oxide (NO) as well as improving Ca^2+^-handling of cardiomyocyte in STZ-induced diabetic rats.

Tissue kallikrein converts kininogen to the peptide hormone bradykinin and kallidin (in humans) or kallidin-like peptide (in rodents) (Campbell et al., 2010). Binding of bioactive bradykinin to bradykinin receptor type I (B1R) and/or B2R stimulates the production of NO and prostaglandins (Harris et al., 2001). Previous studies have demonstrated that the constituent parts of the KKS are locally expressed in the heart (Nolly et al., 1992), and a down-regulated cardiac tissue kallikrein level was found in STZ-induced diabetes (Sharma and Kesavarao, 1996; Tschope et al., 1999). Several researchers have claimed that activating the KKS with gene transfer approaches can improve cardiac function in animal models of DCM (Tschope et al., 2004; Montanari et al., 2005; Tschope et al., 2005). In addition, transgenic rats overexpressing the human tissue kallikrein gene have reduced isoproterenol-induced cardiac hypertrophy and fibrosis, and these protective effects are abolished by icatibant (Silva et al., 2000), which is a specific antagonist of B2R. All these findings indicate a potential protective role of the KKS in DCM. Our study, for the first time, demonstrated that exogenous PKK protected against DCM. Gene therapy technology is difficult and expensive to apply to clinical treatment, while PKK is already available for clinical application in China and Japan. Therefore, exogenous kallikrein supplementation might be promising in treating DCM.

Oxidative stress plays a critical role in the DCM (Wang et al., 2012). It is difficult to determine the production of peroxynitrite (ONOO-), a destructive free radical oxidant. However, nitrotyrosine is a detectable marker for indirect measuring ONOO- (Ischiropoulos, 1998). In our present research, the protein accumulation of nitrotyrosine was increased in the DM group compared with the NDM group, whereas it was decreased by PKK treatment. The GSH/GSSG ratio (GSH/GSSG) is another prominent parameter for maintaining oxidative stress balance (Chepelev et al., 2013). The myocardial GSH/GSSG in DM group was decreased compared with the NDM group, but PKK injections did not reverse this change significantly. GSH/GSSG may not be the main mechanism mediated the beneficial effects of PKK on DCM. The protective mechanisms of KKS against reactive oxygen species damage are capable of eradicating free radicals and preventing them from causing deleterious effects under physiological conditions, including a number of enzymatic and non-enzymatic antioxidants (Elnakish et al., 2013, 2015). Nrf2 plays a central role in DCM in response to oxidative damage (He et al., 2009; Bai et al., 2013). This observation prompted us to measure the expression of antioxidative genes. Our results showed that Nrf2, SOD, CAT, GR, and GPx-1 were downregulated in diabetic rats compared with the NDM group. Conversely, the administration of PKK increased the expression of these genes in the heart of diabetic rats. In addition, Kayashima et al. (2012) reported that KKS protects against oxidative stress and organ damage in the heart. Thus, PKK treatment could abolish oxidative stress imbalance in myocardial tissue of diabetic rats.

Many studies have demonstrated that activated TGF-β1 induces myofibroblast formation and increases extracellular matrix deposition by upregulating collagen synthesis and inhibiting matrix degradation, which leads to myocardial fibrosis (Bujak and Frangogiannis, 2007; Liu et al., 2015; Mewhort et al., 2016). Moreover, kallikrein gene transfer could attenuate myocardial fibrosis (Bledsoe et al., 2003; Tschope et al., 2004). In the present study, the level of TGF-β1 and collagen I increased in the DM group compared with the NDM group, whereas PKK treatment reduced the expression of these two genes. Here, we showed the robust evidence suggesting that PKK protected against diabetes-induced myocardial fibrosis *in vivo*. Inflammation also involves in the onset and development of DCM. Saklani et al. (2016) suggested that TNF-α is essential for DCM. IL-6, CD68, and MCP-1 are also important factors in cardiac inflammation (Guo et al., 2016; Zhang J.S. et al., 2016). Our results displayed increased levels of these inflammatory cytokines in DCM, but they were significantly reduced in DM+PKK group. In the cytoplasm, the IκB-α subunit is kept in an inactive state in the NF-κB multicomponent system. Phosphorylation of IκB-α in response to appropriate stimuli causes degradation and translocation of the NF-κB heterodimer to the nucleus (Karin and Ben-Neriah, 2000; Kumar et al., 2013). As expected, myocardial tissues in diabetic rats displayed decreased levels of IκB-α, thereby activating the NF-κB signaling pathway. PKK treatment significantly recovered the expression of IκB-α. Tschope et al. (2005) also reported that the increase of intramyocardial inflammation was reduced via overexpressing the human kallikrein gene (hKLK1) in a diabetic rat model.

According to previous studies, we speculate that both B1R and B2R mediate the cellular effects of PKK (Silva et al., 2000; Zubakova et al., 2008; Guabiraba et al., 2017). The expression of both B1R and B2R in myocardial tissue was detected in our models. Future experiments with the application of antagonists of bradykinin receptors should be performed to corroborate our speculation.

Several types of cells may be the targets of PKK. We found that both B1R and B2R are expressed in myocardial tissues. Previous studies reported that bradykinin receptors were expressed in endothelial cells (Zhang R. et al., 2016). Binding of bioactive bradykinin to bradykinin receptor type I (B1R) and/or B2R stimulates the production of nitric oxide (NO) and prostaglandins, which can dilate the blood vessels (Harris et al., 2001). In addition, PKK can also reduce inflammation in DCM, which may be through targeting the inflammatory cells (Guabiraba et al., 2017; Sodhi et al., 2018). We found that the CD68 expression in the heart tissues, a marker of macrophage was increased in DM group, while was decreased in DM + PKK group, suggesting that PKK might target the macrophages.

Our STZ-induced diabetic rats largely mimic type 1 diabetes, and thus this model does not precisely represent the type 2 diabetes in humans. Although various pathophysiologic and molecular aspects are common between type 1 and type 2 diabetes, some features may be different, which limits the generalizability of our findings to type 2 diabetes. In our future studies, we need to investigate the efficacy of PKK treatment for the myocardial diastolic dysfunction in DCM by echocardiography *in vivo*. Although the myocardial SERCA2a/PLN ratio and plasma NT-proBNP measured in the present study are reliable markers of heart dysfunction, even early cardiovascular changes, the echocardiography is still the direct identification for DCM. Moreover, we also need to determine the optimal dose of PKK for preserving heart function and inhibiting myocardial remodeling.

## Conclusion

Our present results demonstrate that PKK administration could protect against DCM by regulating myocardial oxidative stress, cardiac fibrosis, inflammation, NO production, and Ca^2+^-handling proteins in diabetic rats. We found no difference between DM + PKK group and DM group in blood pressure and blood glucose levels, indicating the protective role of PKK on DCM was independent of glucose- or blood pressure-lowering effects. Taken together, the application of exogenous PKK is a promising potential therapeutic strategy for DCM. To the best of our knowledge, this is the first study to evaluate the cardio-protective effects of exogenous PKK on DCM.

## Author Contributions

MWu is the main author and contributor of this research. SG and YY contributed to the daily injection of rats. WL captured the TEM images and analyzed the results. MWa, YW, and FZ captured the images and contributed to the analysis of IHC results. ZZ, JHu, MH, QL, JHua, and LZ designed the experiments and interpreted the results of experiments. YL, JHu, WG, and ZZ revised this paper.

## Conflict of Interest Statement

JHua is employed by Changzhou Qianhong Biopharma Co., Ltd. The remaining authors declare that the research was conducted in the absence of any commercial or financial relationships that could be construed as a potential conflict of interest.
