# Supplementary figures and images for: Exogenous Pancreatic Kallikrein Improves Diabetic Cardiomyopathy in Streptozotocin-Induced Diabetes
Source: Front Pharmacol. 2018 Aug 7;9:855. doi: 10.3389/fphar.2018.00855 (PMC6091235; doi:10.3389/fphar.2018.00855)

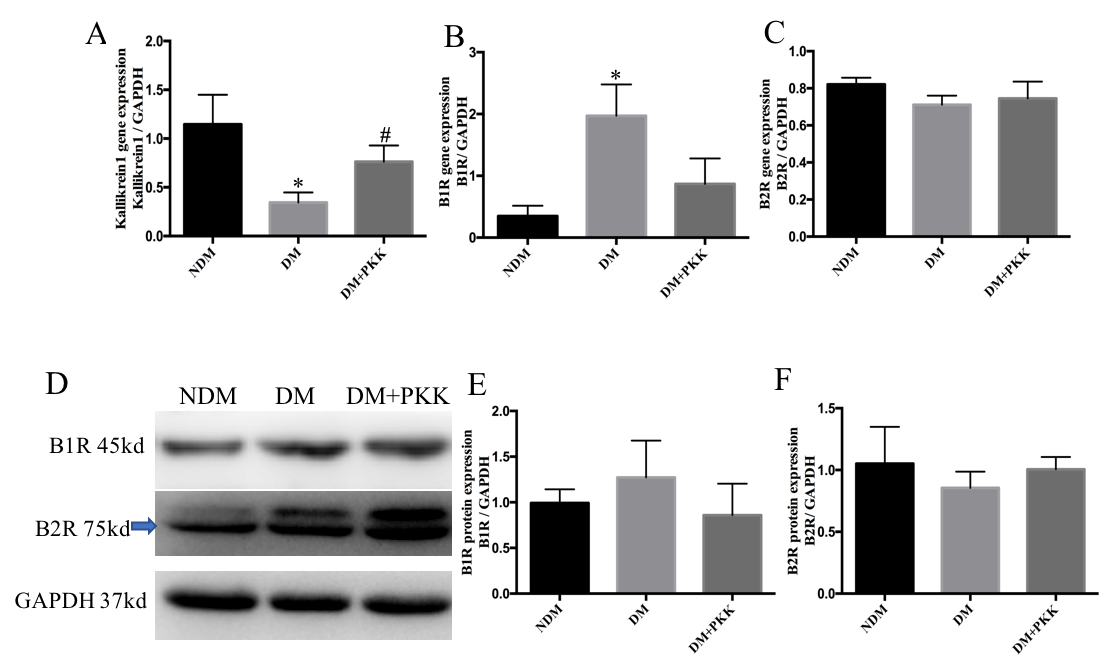

Supplement: Supplementary file 1 [file Image_1.tif]
