# Supplementary material for: Exogenous Pancreatic Kallikrein Improves Diabetic Cardiomyopathy in Streptozotocin-Induced Diabetes
Source: Front Pharmacol. 2018 Aug 7;9:855. doi: 10.3389/fphar.2018.00855 (PMC6091235; doi:10.3389/fphar.2018.00855)
Supplement: Supplementary file 2 [file Table_1.DOC]

**Exogenous pancreatic kallikrein improves diabetic cardiomyopathy in streptozotocin-induced diabetes**

**Supplementary table 1. Sequences of primers used for real-time PCR**

|  | Forward | | Reverse |
| --- | --- | --- | --- |
| GAPDH | | 5’- ACCACAGTCCATGCCATCAC -3’ | 5’-TGCCAGTGAGCTTCCCGTT-3’ |
| Kallikrein1 | | 5’-GTGTGCAGGAGAGTTGGAAGGAG-3’ | 5’-TTGGTGTAGATGGCTGGCATGTTG-3’ |
| B1R | | 5’-TGATCCTCACACTGGTAGCC-3’ | 5’-GGTTCAAGCAGCTGTTGACA-3’ |
| B2R | | 5’-CACTGCAGGGTCTGCAACTA-3’ | 5’-CAGAGTGCTCCTCCCTTGTC-3’ |

B1R, bradykinin receptor type I; B2R, bradykinin receptor type II.
