# Supplementary material for: Exogenous Pancreatic Kallikrein Improves Diabetic Cardiomyopathy in Streptozotocin-Induced Diabetes
Source: Front Pharmacol. 2018 Aug 7;9:855. doi: 10.3389/fphar.2018.00855 (PMC6091235; doi:10.3389/fphar.2018.00855)
Supplement: Supplementary file 3 [file Table_2.DOC]

**Supplementary data 1 KKS in the myocardial tissues of STZ-induced diabetic rats.**

(A) The mRNA expression of Kallikrein 1 gene. (B) The mRNA expression of B1R gene. (C) The mRNA expression of B2R gene. (D) The protein expression of B1R and B2R using western blot. (E) The protein quantitation of B1R. (F) The protein quantitation of B2R. Each sample was repeated for three times. Values are mean ± SEM, n = 6 for per group; *P<0.05 versus NDM group; #P< 0.05 versus DM group.
